# Beyond allostery: Catalytic regulation of a deoxyribozyme through an entropy-driven DNA amplifier

**DOI:** 10.1186/1759-2208-1-13

**Published:** 2010-10-01

**Authors:** Grace Eckhoff, Vlad Codrea, Andrew D Ellington, Xi Chen

**Affiliations:** 1 Institute for Cellular and Molecular Biology, University of Texas at Austin, Austin, Texas 78712, USA; 2 Department of Chemistry and Biochemistry, University of Texas at Austin, Austin, Texas 78712, USA

## Abstract

The programmability and replicability of RNA and DNA have respectively enabled the design and selection of a number of allosteric ribozymes and deoxyribozymes. These catalysts have been adapted to function as signal transducers in biosensors and biochemical reaction networks both *in vitro* and *in vivo*. However, allosteric control of nucleic acid catalysts is currently limited by the fact that one molecule of effector (input) generally regulates at most one molecule of ribozyme or deoxyribozyme (output). In consequence, allosteric control is usually inefficient when the concentration of input molecules is low. In contrast, catalytic regulation of protein enzymes, as in protein phosphorylation cascades, generally allows one input molecule (e.g., one kinase molecule) to regulate multiple output molecules (e.g., kinase substrates). Achieving such catalytic signal amplification would also be of great utility for nucleic acid circuits. Here we show that allosteric regulation of nucleic acid enzymes can be coupled to signal amplification in an entropy-driven DNA circuit. In this circuit, kinetically trapped DNA logic gates are triggered by a specific sequence, and upon execution generate a peroxidase deoxyribozyme that converts a colorless substrate (ABTS) into a green product (ABTS^•+^). This scheme provides a new paradigm for the design of enzyme-free biosensors for point-of-care diagnostics.

## Findings

A variety of functional nucleic acids have been engineered over the past two decades, including not only simple binding elements (aptamers [1,2]) and catalysts (ribozymes [3] and deoxyribozymes [4]), but also more ‘intelligent’ molecular parts, such as aptamer beacons and allosteric ribozymes that can sense biomolecules [5,6], process molecular information [7,8], and regulate biochemical systems [9]. However, most regulatory nucleic acid elements are based on allosteric control, which has a fundamental limitation: one input molecule generally yields only one output molecule. Such stoichiometric or sub-stoichiometric regulation is often insufficient for effective metabolic regulation or diagnostic signal transduction, especially when the concentrations of input molecules are low.

In contrast, natural catalytic cascades, such as the phosphorylation of proteins by kinases, readily amplify low input signals. Although in principle ribozymes and deoxyribozymes could participate in similar cascades as catalysts [10–12], no generalizable method for implementing such cascades has yet been established. On the other hand, DNA and RNA can catalyze chemical reactions not only by forming intricate tertiary structures, but also by simply forming Watson-Crick base pairs. In fact, by serving as a hybridization template, DNA can control and catalyze a wide range of chemical reactions [13], some of which can yield products capable of regulating downstream reactions. More recently, Zhang and coworkers have designed a scheme for highly efficient, enzyme-free, entropy-driven catalytic reactions that relies only on the dynamic hybridization of DNA strands [14–17]. Because of its chemical simplicity, this scheme is expected to allow the development of enzyme-free DNA circuits substantially more complex and robust [18] than previous enzyme-dependent examples [19–22]. Similar strand displacement-based schemes using DNA hairpins as substrates have also been devised [23,24]. Moreover, it has been shown that the principles used to construct aptamer beacons and allosteric ribozymes [5] can also be applied to these schemes in order to create DNA circuits that use molecules other than nucleic acids as input signals [23].

While DNA circuits may prove useful as diagnostics, strand-exchange reactions have so far been monitored by sophisticated analytical devices. As an example of the possibilities available for signal transduction, we convert one molecule of input DNA to multiple molecules of a peroxidase deoxyribozyme [12,25] that in turn converts the colorless substrate 2,2’-azino-bis(3-ethylbenzthiazoline-6-sulphonic acid) (ABTS) into the readily observed green product ABTS^•+^. We also demonstrate that an entropy-driven catalytic DNA circuit can function as a generic signal amplification module that allows lower concentrations of input molecules to control the production of higher concentrations of deoxyribozymes, and thus exceed the limit of stoichiometric allosteric control. The system is qualitatively sensitive to mismatches in the input nucleic acid, suggesting that the combined entropy-driven strand exchange and allosteric deoxyribozyme circuit might facilitate the future development of enzyme-free, point-of-care diagnostic assay system [26–28].

As a starting point for the development of a joint entropy-driven strand exchange and allosteric deoxyribozyme circuit, we began with the one-step, entropy-driven DNA catalytic circuit shown in Figure 1A, similar to that developed by David Zhang and Erik Winfree [16]. The functional segments of each DNA strand are called ‘domains’ and are designated by numbers. Each domain is further divided into two sub-domains: a 6-nt-long toehold [29,30] sub-domain (*t*), and a 14~15-nt-long branch migration sub-domain (*b*). The sequences for all sub-domains are summarized in Table 1.

In this circuit, the amplification module contains a **Gate** duplex at 100 to 200 nM, and a **Fuel** strand at a higher concentration (300 nM to 1 μM); this will allow the entropy-driven execution of the circuit. The **Gate** duplex in turn consists of a **Dock** strand and an **Invader** strand. Although in principle the **Fuel** can displace the **Invader** from the **Gate**, this reaction is very slow due to the stability of DNA duplex. However, in the presence of even low concentrations of an input (the **Trigger** strand), the sub-domain *1t* at the 3’ end of the **Trigger** can bind the toehold sub-domain 1t* at the 5’ end of the **Dock**, and initiate a branch migration reaction in which the **Trigger** displaces the Invader from the **Dock**. After the **Invader** dissociates, a **Fuel** strand can interact with the **Dock** via the sub-domain *2t* at its 5’ end, and initiate a backward branch migration to displace, and thus recycle, the **Trigger** (with the concomitant formation of the **Waste_1** duplex). The recycled **Trigger** can then catalyze another cycle of exchange between the **Invader** and the **Fuel** on the **Dock**. Thus, the net reaction of these two processes is the reversible reaction **Gate + Fuel = Invader + Waste_1**, where the **Trigger** serves as a catalyst to accelerate this otherwise very slow reaction, and thereby reach an equilibrium where a substantial amount of **Invader** is released.

The product of the amplification module, namely the **Invader** strand, can then enter the allosteric deoxyribozyme module (Figure 1B) which consists solely of a **Reporter** duplex. The reporter duplex is formed by the hybridization of the **DNAzyme** strand and the **Blocker** strand. The **DNAzyme** strand has a catalytic domain 3 (colored dark purple in Figure 1B) that can fold into a *G*-quadruplex and exhibit peroxidase activity in the presence of hemin. However, the activity of the deoxyribozyme is inhibited by hybridization to the **Blocker** strand in the **Reporter** duplex. The **Invader** strand produced by the amplification module can displace the **DNAzyme** from the **Reporter** duplex with the concomitant formation of the **Waste_2** duplex. Only upon **Invader**-mediated displacement can the **DNAzyme** strand undergo a conformational change and fold into an active *G*-quadruplex conformation and (in the presence of hemin and H_2_O_2_) oxidize the colorless substrate ABTS to produce the readily observed green product ABTS^•+^. Thus, the overall function of the combined circuit is to amplify one molecule of **Trigger** into multiple molecules of *G*-quadruplex deoxyribozyme and colored product.

In order to assay the functionality of the amplification module we monitored the formation of **Invader** in real-time. The **fReporter** duplex (Figure 2A) was synthesized with a FAM-labeled **fDNAzyme** strand and a quencher-labeled **qBlocker** strand (Figure 2A). Upon addition of Invader the **fDNAzyme** should be displaced from the **qBlocker**, resulting in a stoichiometric (not catalytic) increase in fluorescent signal. Although 100 nM of **fReporter** showed some fluorescent background (defined as 1 RFU) due to incomplete quenching, the addition of **Invader** increased the fluorescence signal up to 2.5 RFU at saturating concentrations (≥ 100 nM, data not shown). In contrast, the addition of 100 nM **Gate** (with or without 1 μM **Fuel**; Figure 2B, blue trace and green trace, respectively) to the **fReporter** caused only a small increase in fluorescent signal (a process commonly called ‘circuit leakage,’ see **Section 2** of [Additional file 1]), likely due to the presence of small amounts of free **Invader**, to the displacement of **fDNAzyme** by the **Gate** duplex in a toehold-independent manner, and/or to the uncatalyzed exchange between the **Invader** and the **Fuel**. However, addition of 5 to 20 nM of **Trigger** in the presence of **Fuel** substantially accelerated the release of **Invader** in a dose-dependent manner, as shown by the much faster increase in fluorescent signal (Figure 2B, red traces). To confirm that the **Trigger** was in fact being recycled to achieve amplification, we carried out the same reaction in the absence of **Fuel** (Figure 2C). As expected, although there were initial bursts of fluorescence signal due to the stoichiometric or sub-stoichiometric displacement of **Invader** by the **Trigger**, the signal did not increase steadily (compare red and magenta traces in Figure 2C).

Figure 2D directly compares the extent of reaction between different samples after 1 hr. Notably, the increase of fluorescent signal by 5 nM **Trigger** in the presence of **Fuel** was ~0.3 RFU (comparing samples #3 and #4), which corresponded to the production of ~20 nM of **Invader**. Thus, in the context of the circuit as a whole 5 nM **Trigger** presumably produced 20 nM **Invader**, and thus the original signal was amplified by (20 nM/5 nM =) 4-fold within an hour. While this value is relatively small compared to other methods, it should be noted that the extent of amplification was primarily limited by the high, uncatalyzed background reaction which in 1 hr produced a signal equivalent to that produced by ~25 nM **Invader**. This background can be tuned by increasing the purity of the **Gate** duplex (see **Section 2** of [Additional file 1]), and by ‘clamping’ and ‘padding’ the duplex to decrease non-specific strand exchange [24].

More important to the function of this chemical system than amplification alone was the ability to modularly generate an optical signal, and the ability to program mismatch discrimination for the **Trigger** input. The allosteric deoxyribozyme module was adapted to the whole circuit shown in Figure 1 by mixing all of the DNA species together in 10 μL volume at 10× final concentration for 30 min, then adding 10 μL of 2 μM hemin followed by 20 min incubation at room temperature (’DNA/hemin mixture’). These sequential incubations should have allowed the formation of deoxyribozymehemin complexes from any **DNAzyme** strands freed due to the execution of the circuit. To initiate the detection reaction, 80 μL substrate solution containing H_2_O_2_ and ABTS was added to the DNA/hemin mixture, making a final volume of 100 μL. The final concentrations of hemin, H_2_O_2_, and ABTS were 200 nM, 2 mM and 2 mM, respectively, and a colorimetric signal could be easily read within 15 minutes. To ensure that signal could be easily detected by eye, 200 nM of **DNAzyme** strand was shown to exhibit strong peroxidase activity in the presence of 200 nM hemin (Figure 3, sample #5), while 200 nM of **Reporter** duplex was inactive under the same conditions (Figure 3, sample #1).

The combined chemical system could thus be assayed for the ability to detect input **Trigger** strands. Notably, in the presence of **Gate** and Fuel, as little as 25 nM of **Trigger** was able to produce sufficient peroxidase activity for visual observation of ABTS^•+^ formation after only 15 min of reaction (Figure 3, sample #6). Observed peroxidase activity further increased when the **Trigger** concentration was increased to 50 nM (Figure 3, samples #7~9). The circuit could be shown to be dependent upon both **Trigger** and **Fuel**. In the absence of **Fuel**, 50 nM of **Trigger** alone did not yield sufficient peroxidase activity for visual observation (Figure 3, sample #13). In the absence of **Trigger**, 200 nM of **Gate** only restored a small fraction of peroxidase activity even with the presence of 500 nM of **Fuel** (Figure 3, sample #2 and #3). As a positive control, the addition of 200 nM of **Invader** to 200 nM of **Gate** and 200 nM of **Reporter** almost completely restored peroxidase activity (Figure 3, compare sample #4 with sample #5).

Zhang and Winfree have shown that single-nucleotide mutations, insertions, and deletions in the catalyst strand can result in 10- to 300-fold reductions in its efficiency in catalyzing strand exchange [17]. These reductions in signal observed with single-nucleotide mismatches are comparable to other well-developed DNA sensors [26–28,31–33]. Consistent with these findings, we observed that when a single nucleotide was changed in the toehold region (Figure 3, inset), the mutant **Trigger** did not yield any peroxidase activity above background even at 150 nM concentration (Figure 3, sample #10~12).

In summary, by coupling an entropy-driven DNA circuit with deoxyribozyme catalysis we created a catalytic cascade akin to phosphorylation-based protein regulation in biology. Optimization of circuit design and purification methods (see **Section 2** of [Additional file 1]) coupled with cascaded and autocatalytic amplification schemes [14,24] should greatly improve circuit performance. Overall, these results suggest that entropy-driven DNA circuits coupled with colorimetric readouts could lead to new point-of-care nucleic acid detection methods, including field-based qualitative plus/minus discrimination between mutant and wild-type alleles.

## Figures and Tables

**Figure 1:**
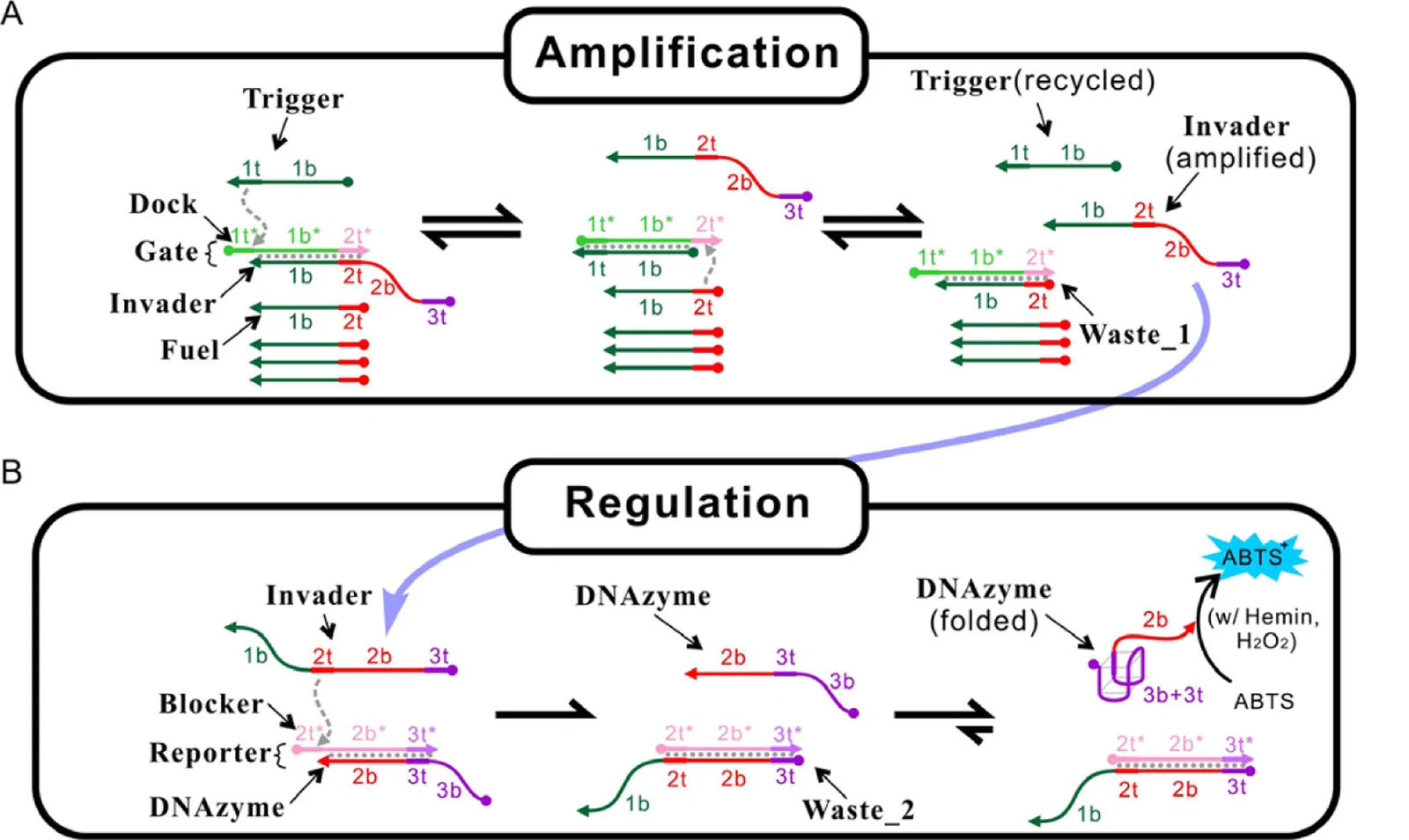
Entropy-driven deoxyribozyme circuit. (A) Scheme for the amplification module, based on an entropy-driven, strand-exchange amplifier. The functional domains within each DNA strand have been color-coded (see Table 1 for the sequence of each domain). Complementary domains are represented by light and dark variation of the same color (i.e., light green pairs with dark green). An asterisk in the names of sub-domains indicates complementarity (i.e., sub-domain 1t* is complementary to sub-domain *1t*). Gray dashed arrows indicate where toehold binding should initiate. Arrows and dots on DNA strands represent 3’ termini and 5’ termini, respectively. (B) Scheme for the reporter module based on an allosteric G-quadruplex peroxidase. The domain containing the G-quadruplex is colored in purple.

**Figure 2:**
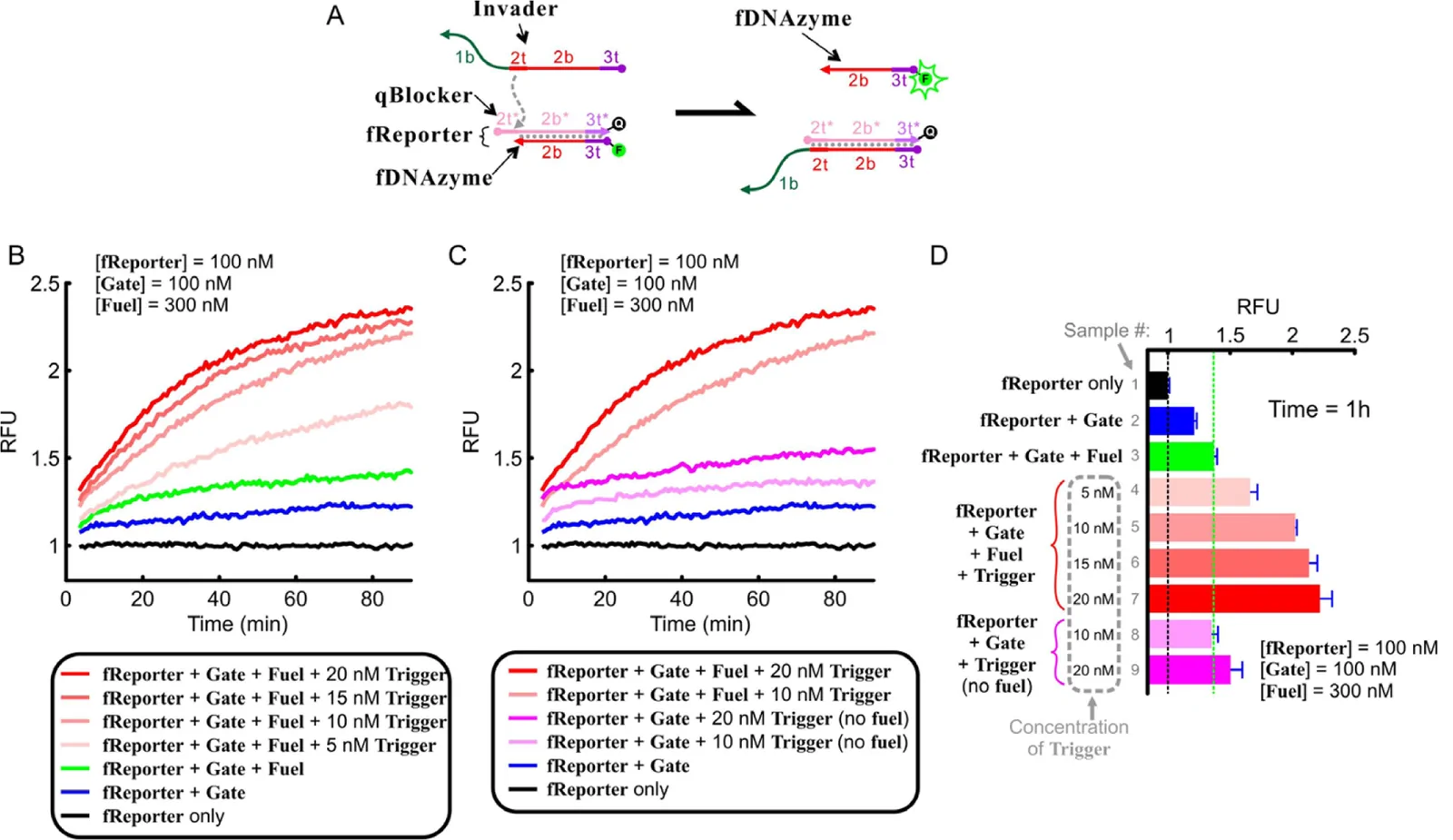
Performance of the amplification module. (A) Scheme for the fluorescence reporter. Color coding is as in Figure 1. (B and C) Kinetics of fluorescent signal accumulation, highlighting the effects of **Trigger** addition (B) and **Fuel** addition (C). (D) Fluorescent signals for all samples at the 1 hr time point. Color coding is the same as in (B) and (C). The residual fluorescent signal due to incomplete qunching of 100 nM **fReporter** was arbitrarily defined as 1 RFU. The error bars represent the standard deviation of the fluorescent signals from two identical reactions that were carried out and measured in two wells of a 384-well plate. Whenever present, the final concentrations of **fReporter**, **Gate** and **Fuel** in these experiments were 100 nM, 100 nM, and 300 nM, respectively. The concentrations of **Trigger** in different reactions were as indicated.

**Figure 3:**
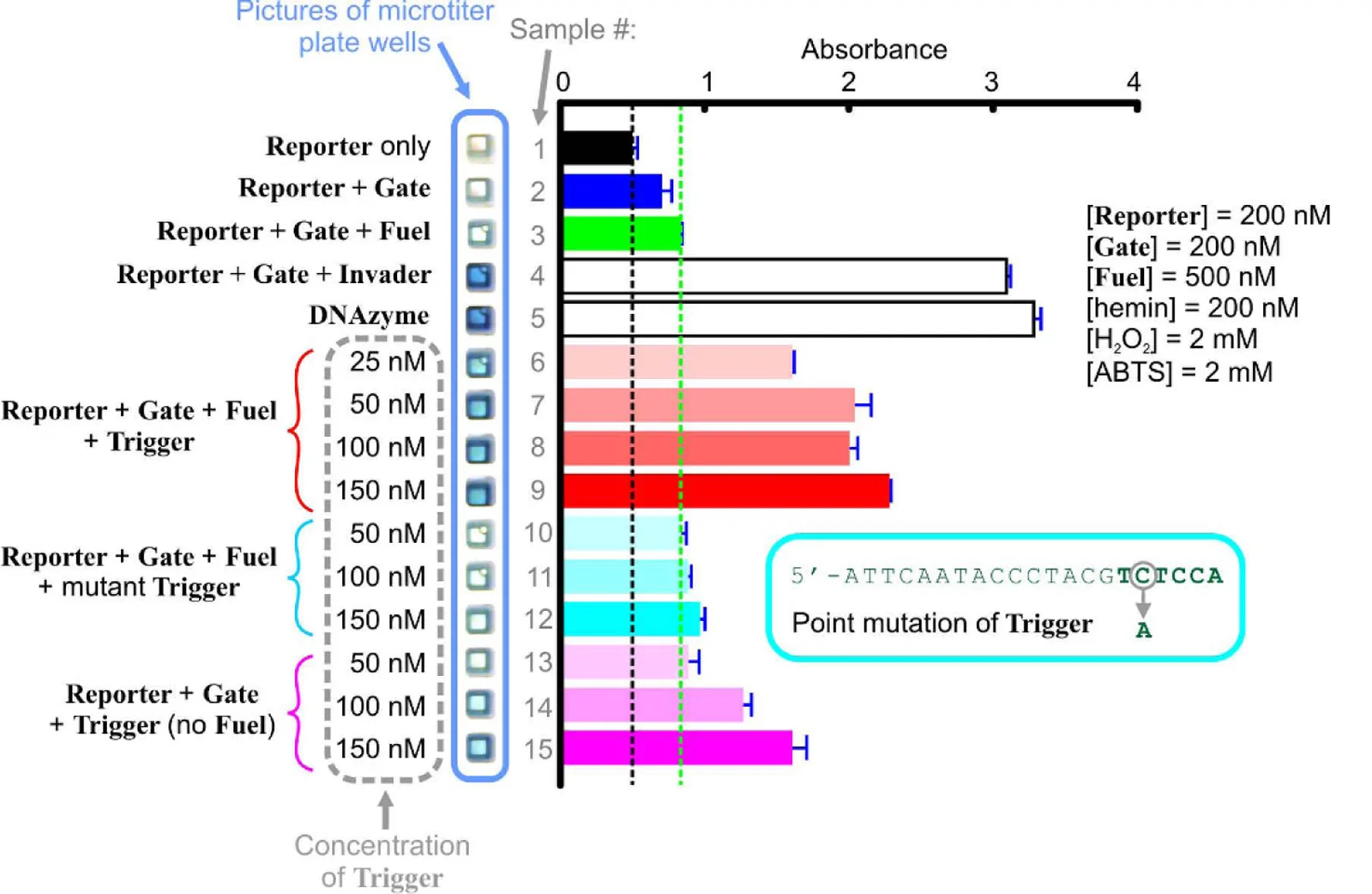
Performance of the entropy-driven deoxyribozyme circuit. Sample numbers (as indicated in the text) are listed next to the bars. Color coding is as in Figure 2D. The pictures of the microtiter-plate wells containing different samples are shown in the blue box. The absorbance readings (at 414 nm) shown in the bar graph were taken 10 min after the H_2_O_2_ and ABTS were added to the DNA/hemin mixture and the pictures were taken 15 min after the H_2_O_2_ and ABTS were added to the DNA/hemin mixture. The inset shows the point mutation in the mutant **Trigger**. The toehold sequence is highlighted in bold. Whenever present, the final concentrations of **Reporter**, **Gate**, **Fuel**, **Invader**, and **DNAzyme** in these experiments were 200 nM, 200 nM, 500 nM, 200 nM, and 200 nM, respectively. The concentrations of **Trigger** and mutant **Trigger** in different reactions were as indicated.

**Table 1:** Sequence of DNA sub-domains used in this work

Sub-domain	Sequence
1t	TCTCCA
1b	ATTCAATACCCTACG
2t	CGTCTC
2b	CGTAAATATTGGCG
3t^†^	GAGGGA
3b	CTGGGAGGGAGG

† The domain 3t of fDNAzyme is AGGGA instead of GAGGGA, in order to avoid quenching of the FAM by the adjacent 5’ G.
